# Rapid and cost-effective generation of single specimen multilocus barcoding data from whole arthropod communities by multiple levels of multiplexing

**DOI:** 10.1038/s41598-019-54927-z

**Published:** 2020-01-09

**Authors:** Guillemette A. de Kerdrel, Jeremy C. Andersen, Susan R. Kennedy, Rosemary Gillespie, Henrik Krehenwinkel

**Affiliations:** 1 Department of Environmental Sciences, Policy and Management, University of California Berkeley, Mulford Hall, Berkeley, California, USA; 2 Department of Biogeography, Trier University, Trier, Germany; 3 Okinawa Institute of Science and Technology, Onna, Japan

**Keywords:** Ecological genetics, Taxonomy

## Abstract

In light of the current biodiversity crisis, molecular barcoding has developed into an irreplaceable tool. Barcoding has been considerably simplified by developments in high throughput sequencing technology, but still can be prohibitively expensive and laborious when community samples of thousands of specimens need to be processed. Here, we outline an Illumina amplicon sequencing approach to generate multilocus data from large collections of arthropods. We reduce cost and effort up to 50-fold, by combining multiplex PCRs and DNA extractions from pools of presorted and morphotyped specimens and using two levels of sample indexing. We test our protocol by generating a comprehensive, community wide dataset of barcode sequences for several thousand Hawaiian arthropods from 14 orders, which were collected across the archipelago using various trapping methods. We explore patterns of diversity across the Archipelago and compare the utility of different arthropod trapping methods for biodiversity explorations on Hawaii, highlighting undergrowth beating as highly efficient method. Moreover, we show the effects of barcode marker, taxonomy and relative biomass of the targeted specimens and sequencing coverage on taxon recovery. Our protocol enables rapid and inexpensive explorations of diversity patterns and the generation of multilocus barcode reference libraries across whole ecosystems.

## Introduction

The world is changing at an unprecedented rate. Small-scale changes at a local scale can propagate and lead to a state shift of the entire system^1^. To understand how shifts in ecosystem properties occur, detailed quantification of change over time has become an important objective of biodiversity research^2^. Traditionally, such biodiversity quantification was based on identification of specimens by trained taxonomic experts, and therefore has generally been targeted only at specific lineages of organisms. But understanding state shifts across an entire ecosystem requires quantifying diversity across all taxa within that system, a task that has remained challenging because of the sheer numbers and diversity of organisms involved.

One approach to large-scale species identification that has the potential to reduce the time required to provide specimen identifications is molecular barcoding^3^. Under this approach, specimens are identified using short PCR amplicons. In recent years, DNA barcoding has greatly profited from the emergence of next generation sequencing (NGS) technology. Current NGS platforms allow the parallel generation of barcodes for hundreds of specimens, at a fraction of the cost of Sanger sequencing^4^. But even using NGS approaches, the barcoding of large numbers of samples is laborious and expensive, as it involves separate DNA extractions and PCRs for every specimen. While it may be possible to reduce some of these costs by avoiding DNA extractions and using direct PCR^5^, the utility of this method is currently limited to certain taxonomic groups^6^. Molecular barcoding thus reaches its limitations when researchers seek to characterize large biodiversity collections, which often exceed tens of thousands of specimens.

A possible solution to the problem of generating barcode sequence data for large biodiversity collections is provided by metabarcoding^7,8^, the amplification and sequencing of pooled community samples. This approach allows for the characterization of species compositions of whole ecosystems at greatly reduced cost and effort. As a result, metabarcoding is rapidly increasing in popularity. However, it also has several important drawbacks^9,10^. For large biodiversity studies, these include but are not limited to: (1) The efficiency of metabarcoding depends on system-specific factors, which are often unknown *a priori*^11^. This necessitates extensive optimization before large scale community analyses can be performed. (2) Metabarcoding relies on well-developed reference libraries. However, as many taxa and geographic regions are underrepresented in barcode databases^12^, it is often necessary to generate these reference libraries before meaningful metabarcoding can be performed^13^. (3) It is impossible to link individual specimens and phenotypes to recovered sequences, as the taxonomic identity of specimens in metabarcoding is assigned purely by comparisons to sequence reference databases. However, such phenotypic information is essential for many ecological analyses^6^. And (4) The inability to link specimens to sequences means that independently generated sequences from multiple non-overlapping loci cannot be assigned to the same specimen through metabarcoding. As a result, only short single-locus analyses are possible, which limits phylogenetic and taxonomic resolution of metabarcoding^14,15^. This latter point is particularly important as barcoding analysis should ideally be based on information from multiple independent loci^16^. As mitochondrial sequences can be prone to over-differentiation compared to the nuclear genome^17^, nuclear data in particular are important to back up mitochondrial inferences of divergence^18^.

**Figure 1:**
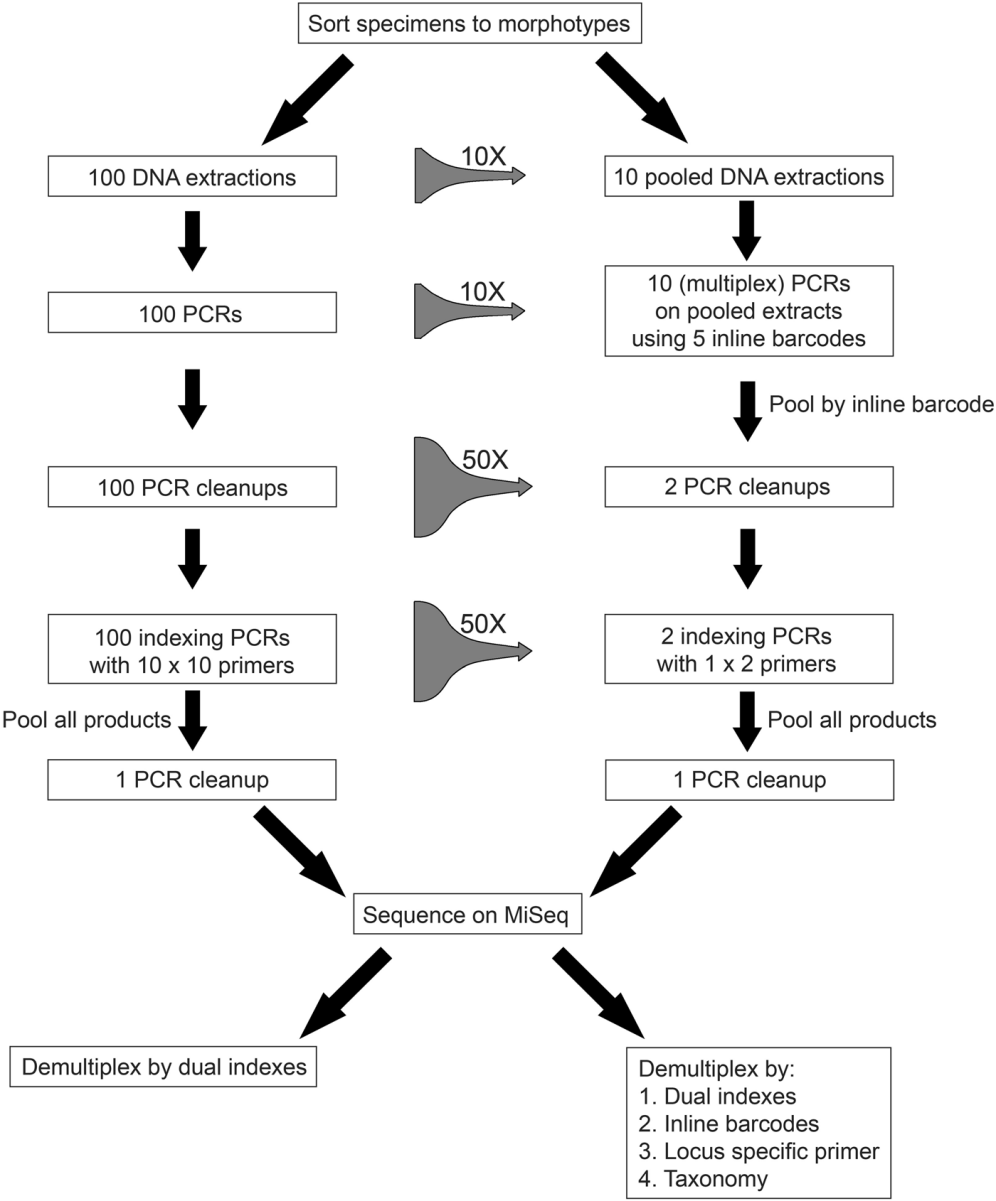
Summary of library preparation workflow in our experiment (right) and a comparable workflow for commonly used amplicon sequencing protocols (left), assuming barcode sequences for 100 specimens have to be generated. The middle column shows the fold change in processing cost and workload for the different steps.

Therefore, the problem remains of how best to generate multilocus barcoding data for massive numbers of organisms, while still being able to assign sequence information to specimens and phenotypes, in a manner that is time- and cost-efficient. Here we propose a solution that considerably reduces processing effort and cost per sequence by integrating the sample multiplexing ability of the Illumina sequencing platform with multiplex PCR and bulk DNA extraction (see Fig. 1). Our approach includes: (1) Two levels of sample indexing: In addition to the use of dual indexes incorporated in a second round PCR^19^, we use inline barcodes in the first round PCR^20^. This allows for the pooling of PCR products pre-indexing and considerably reduces the necessary number of primers and PCRs. (2) Multiplex PCRs are used to generate multilocus datasets for specimens with the locus-specific primer sequences serving as additional barcodes to demultiplex loci^16^. (3) Pooled extractions and PCRs: Public databases are commonly used for the matching of DNA barcode sequences to reference taxa from across the tree of life^21^, often allowing matches to relatively low taxonomic level. DNA extractions and PCRs can thus be performed with pooled specimens. Sequences can be assigned back to their respective specimens if the taxonomic composition of the pool is known and pooled specimens are not too genetically similar.

**Figure 2:**
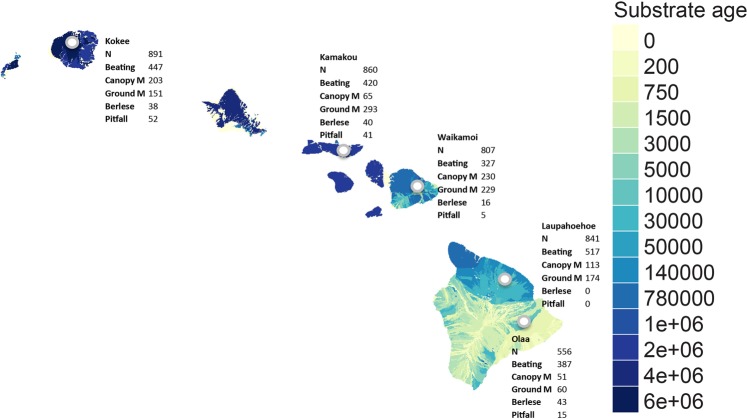
Location of sampling sites across different islands and substrate ages of the Hawaiian Archipelago. The number of specimens included in our analysis for each site and trapping method is shown.

We present a laboratory and computational workflow from specimens to sequences. We then test this protocol by generating ecosystem wide barcode information for a comprehensive collection of nearly 4,000 Hawaiian arthropods (insects, crustaceans, arachnids and myriapods), representing 14 different orders. These specimens were collected from five sites on four islands using five different trapping methods (Fig. 2). With this dataset, we test the effects of taxonomy, sampling method, relative body size, read coverage, and number of multiplexed samples on barcode sequence recovery success. In addition, we explore the taxonomic utility of different genetic markers in comparison to the standard barcode marker (COI), and test how many markers are needed to achieve a complete or near complete recovery of all taxa in a given community. Finally, we provide an overview of taxonomic richness for various taxa and the utility of different trapping methods to recover diversity among Hawaiian arthropods.

## Methods

### Specimen collection and taxonomic sorting

Arthropod samples were collected from five sites of increasing substrate age on four islands of the Hawaiian Archipelago (Fig. 2). Sites were chosen to be as similar as possible in terms of rainfall, elevation, forest type (*Metrosideros* canopy), and minimal evidence of invasion by nonnative species^22^. At each site, an exhaustive sampling protocol using five different trapping methods was employed, aimed at collecting a complete representation of local diversity of arthropods. Soil arthropods were collected by Berlese and pitfall traps; flying insects were collected by canopy and ground Malaise traps; and plant-associated insects were collected by beating vegetation for 10 minutes at each site and collecting specimens from the beat sheets. All samples were taken to the University of Hawaii at Hilo, where they were stored in 99% ethanol at −20 °C and subsequently shipped to the University of California Berkeley.

All specimens from each site and trapping method were sorted to order, and then to finer taxonomic classifications using a morphotype-based approach similar to Emerson *et al*.^23^ (i.e., distinct differences in size, color, shape, or other specific characters relevant to a given order). At least one representative specimen of each morphotype was selected for molecular analyses (though when possible, five specimens from each morphotype were selected to explore within-morphotype variation). Each selected specimen was photographed, and body length (head-abdomen) was measured to 0.5 mm accuracy using graph paper under a stereo microscope. We excluded mites (Acari) and thrips (Thysanoptera), as they will be used for more detailed morphological analysis in the future. In total, 3,973 specimens from 14 orders were included in the analyses.

### DNA extraction and sequencing library preparation

Pooled DNA extractions were performed by combining specimens belonging to different orders. Between four and ten specimens were pooled into a single well of a 1 ml 96 well block plate (Fisher Scientific, Hampton, NH, USA), making a total of 6 plates each containing between 386 and 834 specimens. Total genomic DNA was extracted from each pool of specimens as follows: 400 µl Cell Lysis Solution (Qiagen, Hilden, Germany) and a 5 mm stainless steel bead (OPS Diagnostics, Metuchen, NJ, USA) were added to each well and the plates were sealed with a reusable silicone seal (Fisher Scientific). The samples were mechanically disrupted using a 2010 Geno/Grinder® (SPEX® Sample Prep, Metuchen, USA) at 1,300 hz for 1.5 min, and then lysed at 56 °C overnight. Purified DNA was precipitated by adding 1 volume of isopropanol and isolated using magnetic beads (Bioneer, Kew East VIC, Australia) on a pipetting robot (Beckman Coulter, Indianapolis, USA). DNA extracts were eluted in 50 µl of TE buffer and a 1:4 dilution was used for subsequent PCRs.

Two separate experiments were conducted using primer combinations presented in Table 1. These primer combinations were selected from a large set of primers previously tested for consistent and efficient amplification of a wide range of arthropods^13,16^. For both experiments, PCRs were run using the Qiagen Multiplex PCR kit with 25 cycles according to the manufacturer’s protocol (Qiagen, Hilden, Germany) in 10 µl volumes.

**Table 1:** Targeted loci and primer combinations used in this study.

Marker	Gene	Forward	Sequence 5′-3′	Reverse	Sequence 5′-3′
COIA	COI	ArF1^15^	GCNCCWGAYATRGCNTTYCCNCG	Fol-degen-rev^7^	TANACYTCNGGRTGNCCRAARAAYCA
COIB	COI	mlCOIintF^14^	GGWACWGGWTGAACWGTWTAYCCYCC	Fol-degen-rev^7^	TANACYTCNGGRTGNCCRAARAAYCA
18SV1-2	18SrDNA_V1-2	SSU_FO4^41^	GCTTGTCTCAAAGATTAAGCC	SSU_R22^41^	GCCTGCTGCCTTCCTTGGA
18SV6-7	18SrDNA_V6-7	18s_2F^42^	AACTTAAAGRAATTGACGGA	18s_4R^42^	CKRAGGGCATYACWGACCTGTTAT
28SD6	28SrDNA_D6	28s_3F^42^	TTTTGGTAAGCAGAACTGGYG	28s_4R^42^	ABTYGCTACTRCCACYRAGATC
H3	Histone H3	H3aF^43^	ATGGCTCGTACCAAGCAGACVGC	H3Ar^43^	ATATCCTTRGGCATRATRGTGAC

### Experiment 1 – Ecosystem wide barcode analysis

A 450 bp stretch of the COI “barcode” region was amplified for all 3,973 specimens using the reverse primer Fol-degen-rev and the forward primer ArF1. The forward primer was modified with one of six different 6 bp inline barcodes added to the 5′-end. After PCR, the relative DNA concentration for each sample was quantified on an agarose gel and PCR products from all six plates were pooled into a single 96-well plate in approximately equal concentrations (adjusted for the number of specimens in each DNA pool), and then cleaned of residual primers using 1X AMpure XP Beads (Beckman Coulter).

### Experiment 2 – Generation of multilocus barcode data from community samples

We then tested the utility of multilocus barcode markers and the efficiency of multiplex PCRs from pooled extractions, using the plate extracted from 834 individuals. Two sets of PCR amplification were performed. In the first, we amplified a 350 bp stretch of the COI barcode region using the primers mlCOIintF/Fol-degen-rev. In the second, we performed a multiplex PCR of four nuclear DNA fragments that included the D6-region of the 28SrDNA, the V1-2 and the V6-7 regions of 18SrDNA and Histone H3 (Table 1). PCRs for the nuclear and mitochondrial markers were run separately as they required different annealing temperatures (46 °C for COI and 55 °C for the nuclear genes). PCRs were quantified, pooled into a single plate, and cleaned as described above.

Indexing PCRs were then performed with the cleaned PCR products from experiments 1 and 2. Indexing PCRs incorporated forward and reverse barcode primers, each with a unique 8 bp index, to allow for demultiplexing following Illumina MiSeq sequencing^19^. A total of twelve forward and sixteen reverse primers were used to index the two final pooled plates from the two experiments. Products from each indexing PCR were subsequently quantified, pooled, and cleaned as described above. The final library contained amplicon sequences from 3,973 specimens and six different gene fragments, and was sequenced on an Illumina MiSeq (Illumina, San Diego, CA, USA) using V3 chemistry and 2 × 300 bp reads at the Vincent J. Coates Genomics Sequencing Laboratory at the University of California Berkeley.

### Sequence analysis

Raw sequence reads were demultiplexed using bcl2fastq (Illumina) and the paired reads merged using PEAR^24^ with a minimum overlap of 50 bp and a minimum quality score of Q20. The assemblies were quality filtered (≥90% bases with ≥Q30) and converted to fasta format using the FASTX-Toolkit^25^. The six inline barcoded plate samples were demultiplexed using UNIX by identifying and separating sequences starting with the according inline barcode. Similarly, the five different amplicons were demultiplexed by identifying sequences starting and ending with the locus-specific forward and reverse primer sequences. Primers and barcodes were then trimmed off using sed. The resulting sequence files were dereplicated and clustered into operational taxonomic units (3% radius OTUs) using USEARCH^26^ with the -sizeout option utilized to add size annotations to each OTU. The de novo chimera removal tool of USEARCH was used to filter out chimeric sequences.

OTU sequence files were concatenated for each amplicon such that one file contained all sequence information for each separate marker. For protein coding loci, sequences were translated and any sequence with an internal stop codon was removed as a likely pseudogene. Sequences were then compared to those published in NCBI GenBank using the BLASTn search algorithm^27^, using default search parameters and retaining the 10 top-scoring BLAST hits per sequence and the staxid for each match. We used a custom Python script (provided as supplemental material) to obtain the complete taxonomic hierarchy (e.g., kingdom, phylum, order, etc.) of each BLAST match, and the results were used to assign OTUs to their respective orders. Non-arthropod BLAST hits, e.g. nematodes and bacteria, were removed. Sequences with ambiguous assignments (e.g., different taxa matching the same sequence) were further analyzed by aligning them with the properly assigned sequences and building preliminary phylogenies in MEGA^28^. Order was then assigned based on phylogenetic placement. If more than one OTU per well matched the same arthropod order, then we assigned the proper taxonomic ID based on the following: 1) OTU size, because the read abundance of the correct taxon should be significantly (>5 times) larger than that of contaminating sequences^6^, and 2) reference photos of the specimens from the according well, many of which we were able to identify to family or genus based on morphology. This way, we could remove most contaminating sequences.

### Experiment 1 – Ecosystem wide barcode analysis

Using the COI dataset of 3,973 specimens, we tested for specific factors associated with sequence recovery (i.e., the success of generating barcode sequences from mixed samples). We tested for the effects of 1) taxonomy (order), 2) trapping method, 3) the relative body size of specimens in each pool (the proportion of their body size out of the total body size of specimens in a pool), 4) the read depth per sample, and 5) the number of specimens in a pool, using a generalized linear model (GLM) in R^29^. We then performed an OTU clustering with all recovered COI sequences for all separate specimens in USEARCH to estimate OTU richness per order and sampling site. This way, we reduced the total number of specimens to actual taxonomic entities, i.e. clusters. We then compared the recovered OTU richness for each arthropod order to the currently known arthropod species diversity on the Hawaiian Archipelago (Hawaiian Arthropod Checklist^30^). Lastly, we analyzed the recovery of different taxa using the various trapping methods, by comparing the numbers of collected specimens and the numbers of recovered OTUs between these methods. As we employed a highly standardized sampling protocol, the number of specimens and number of OTU clusters likely represent the actual diversity present at each site during the sampling effort and should allow for comparisons between sites and trapping methods. We thus did not rarefy our datasets for the ecological analyses.

### Experiment 2 – Multilocus dataset

Using the multilocus dataset of 834 specimens, we compared the recovery success of specimens between the different amplified markers. We estimated the increase of recovered specimens by using more than one barcode marker and tested how many markers are necessary for an exhaustive recovery of diversity. We also performed OTU clustering for all six amplicons and compared the recovery of OTUs between mitochondrial COI and nuclear markers. We were particularly interested in the comparative performance of these markers in the recovery of taxa and their respective resolution for distinguishing species.

## Results

### Experiment 1 – Ecosystem wide barcode analysis

Illumina MiSeq sequencing resulted in good coverage for 546 of 576 of the sequenced plate wells. 30 samples were excluded due to low sequence coverage of the pool. Pooling did not lead to considerable biases in read recovery, but there was a slight, but significant (Pairwise Wilcoxon test, *P* < 0.05), drop in recovery for Plate 3 (Suppl. Fig. 1A). The absolute number of recovered reads per well and specimen (Table 2), as well as the relative read content, corrected by the specimen’s relative body size (Suppl. Fig. 1B), was quite disparate between arthropod orders (Pairwise Wilcoxon test, *P* < 0.05). Consequently, an increase in the number of orders in a pool led to a more pronounced bias of read abundances between specimens (Suppl. Fig. 1C, Pairwise Wilcoxon test, *P* < 0.05).

**Table 2:** Summary of our barcoding experiment of 3,973 specimens.

Taxon	#Specim.	#Recov.	%Recov.	#OTUs	#Reads	mm	%Simil.
Amphipoda	46	43	93.48%	5	127.12 ± 133.84	6.15 ± 2.51	90.71 ± 7.03
Araneae	474	393	82.91%	68	83.07 ± 97.11	3.23 ± 1.56	97.21 ± 2.87
Blattodea	19	18	94.74%	1	366.78 ± 272.95	5.28 ± 2.98	86.87 ± 0.08
Coleoptera	576	393	68.23%	84	85.75 ± 135.58	4.19 ± 1.99	88.58 ± 4.71
Collembola	192	174	90.63%	9	66.34 ± 64.03	2.66 ± 0.91	95.90 ± 6.12
Diptera	533	440	82.55%	134	149.13 ± 199.62	3.25 ± 1.89	90.58 ± 5.15
Hemiptera	480	429	89.38%	94	188.03 ± 310.76	4.16 ± 1.75	89.87 ± 4.38
Hymenoptera	192	103	53.65%	40	41.07 ± 56.97	3.85 ± 1.75	93.21 ± 6.06
Isopoda	192	104	54.17%	2	74.35 ± 98.83	4.41 ± 2.99	93.70 ± 8.36
Lepidoptera	480	390	81.25%	112	194.90 ± 172.67	5.30 ± 2.15	93.85 ± 2.98
Neuroptera	21	18	85.71%	10	274.56 ± 310.76	7.89 ± 3.36	93.68 ± 3.48
Myriapoda	96	91	94.97%	10	187.28 ± 204.72	—	96.96 ± 6.39
Orthoptera	192	160	83.33%	9	184.83 ± 144.50	3.69 ± 1.67	90.06 ± 1.53
Psocoptera	480	361	75.21%	40	69.28 ± 74.34	2.28 ± 0.41	84.22 ± 1.71

The table presents the number of analyzed (#Specim.) and recovered (#Recov.) specimens and the percent of specimens recovered (%Recov.) as well as the number of OTUs (#OTUs) for each arthropod order. It also shows the average number of reads per sequenced specimen (#Reads), the average body size (mm) and the average percent similarity (%Simil.) to the best BLAST hit ± Stdev for each statistic, respectively.

**Figure 3:**
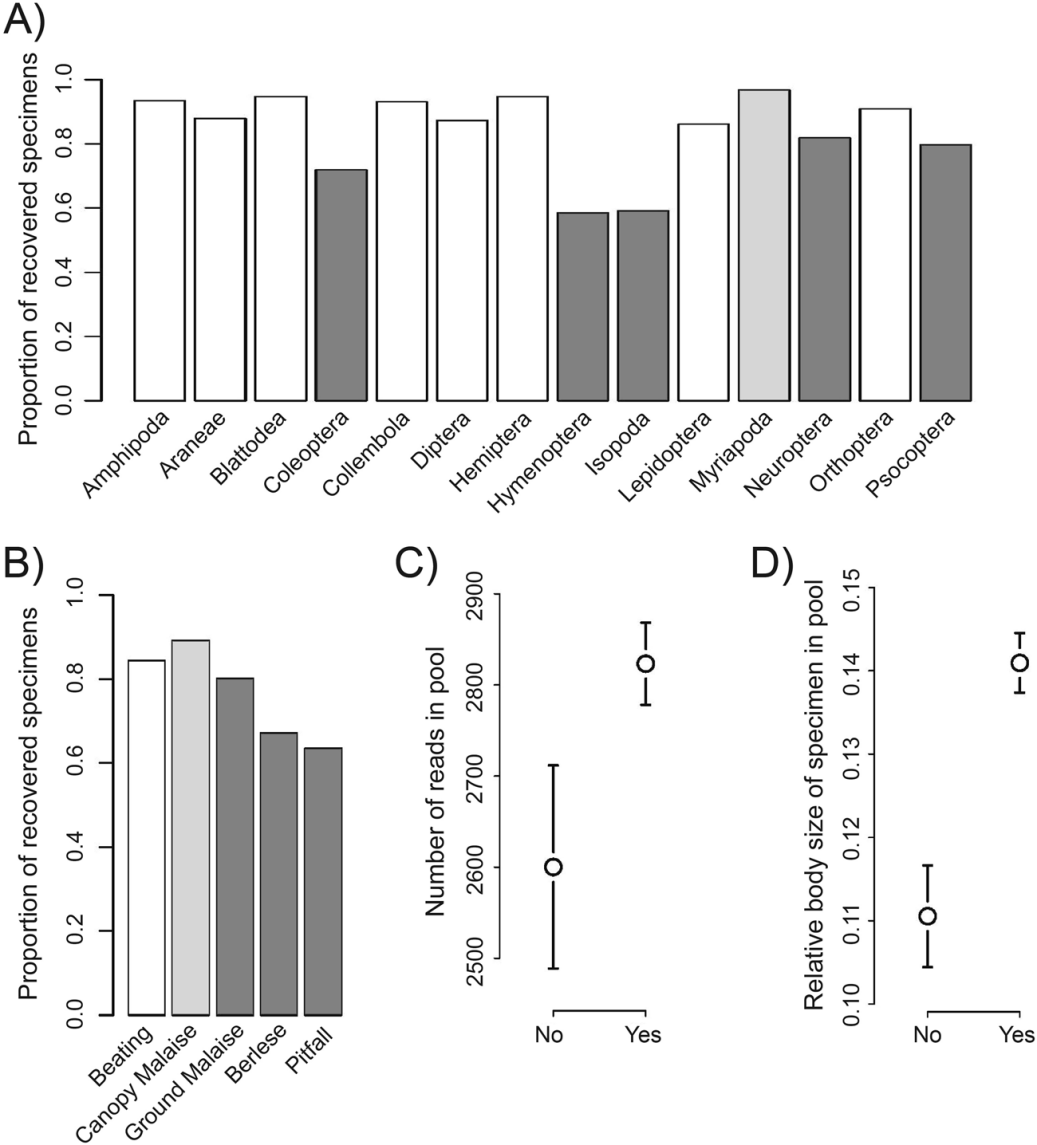
Factors showing a significant association with sequence recovery in our barcode sequencing experiment of 3,973 specimens. (**A**) Proportion of recovered specimens from all 14 arthropod orders. (**B**) Proportion of recovered specimens from five different trapping methods. (**C**) Average read coverage of specimen pools with successful barcode recovery (“Yes”) and those for which we could not recover barcode sequences (“No”). (**D**) Relative body size (mm) among pools of specimens for which we successfully generated barcode sequences (“Yes”) and for those that failed (“No”). Dark grey indicates significantly lower recovery and light grey significantly higher recovery than white.

The recovery of specimens differed significantly between orders, with fewer barcode sequences recovered for Isopoda, Hymenoptera and Coleoptera than other taxa (Fig. 3A, Table 2). The method of collection also had an effect on recovery, with fewer specimens recovered from the two soil sampling methods (pitfall = 64%, Berlese = 67%) than from Malaise traps (canopy Malaise = 89%, ground Malaise = 80%) or beating (84%) (Fig. 3B). The sequencing depth of specimen pools also had a significant effect, with higher sequencing coverage being associated with sequencing success (Fig. 3C). Moreover, the relative body size of specimens in their respective pools was significantly and positively associated with barcode recovery (Fig. 3D). No significant association was found between barcode recovery and the number of specimens in a pool.

**Figure 4:**
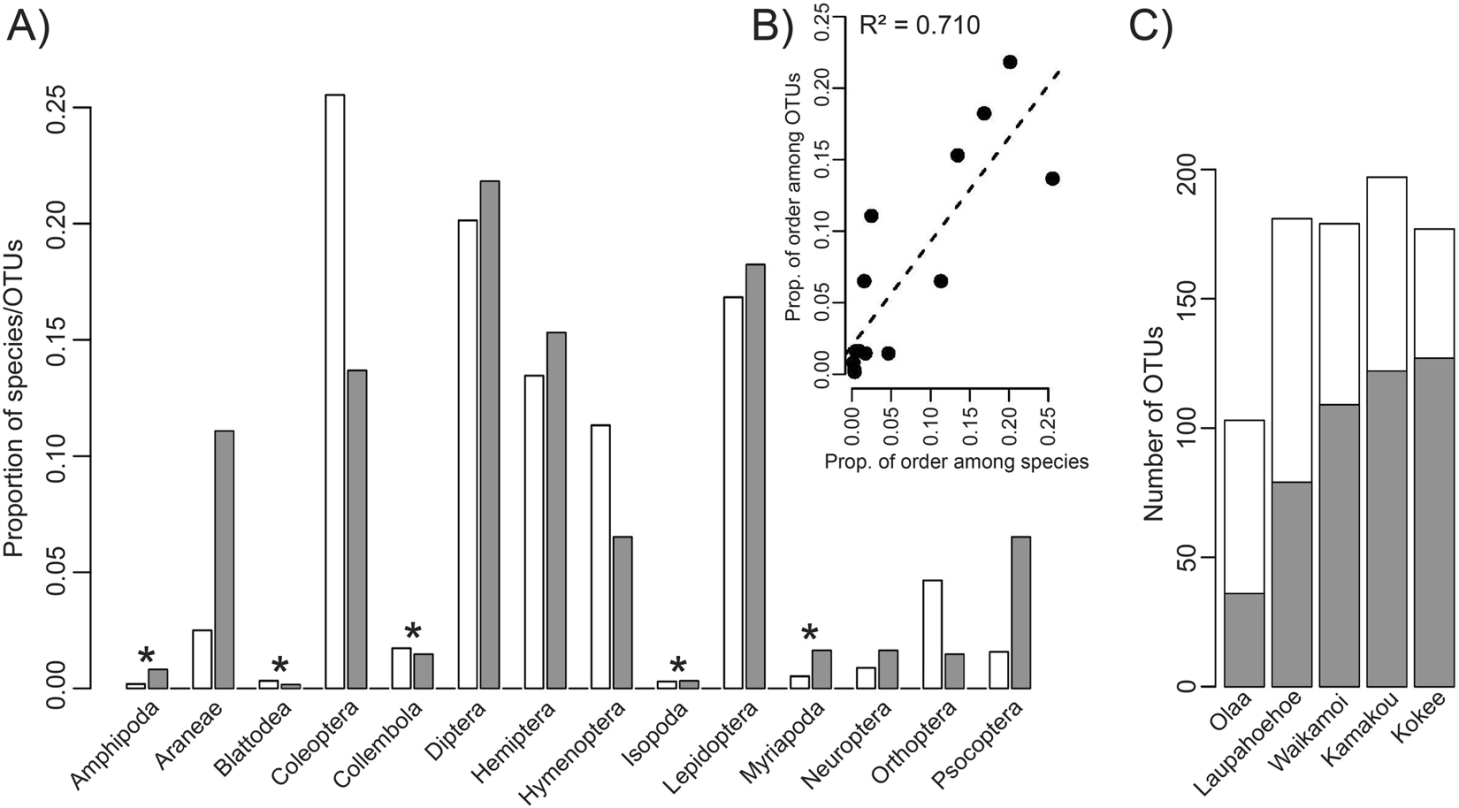
(**A**) Barplot showing the proportion of the 614 COI OTUs recovered from 3,117 arthropod specimens across 14 orders (grey) and the proportion of the 5,720 known Hawaiian species across the same 14 orders (white). A * indicates orders that are dominated by invasive taxa. (**B**) Association of the proportion of all known Hawaiian arthropod species (based on the Hawaiian Arthropod Checklist^30^) and all OTUs across the 14 orders. (**C**) Number of all recovered OTUs per sampling site (white) and number of OTUs that were exclusively found at a single sampling site (grey) across all 14 arthropod orders.

Barcode sequences for 3,117 of the 3,973 specimens were recovered (Table 2), with average similarity to the closest matching sequence in the NCBI database differing significantly between orders (Table 2; Pairwise Wilcoxon Test, *P* < 0.05). At a 3% OTU clustering threshold, the 3,117 recovered specimens fell into 614 OTUs. Most of these OTUs were represented by only a single specimen, leading to highly skewed abundance distributions (Suppl. Fig. 2A). This pattern held true across different arthropod orders (Suppl. Fig. 2B). The number of OTUs was significantly different (χ^2^ test, *P* < 0.05) between orders, ranging from only a single OTU for Blattodea, to 134 for Diptera (Table 2). Most groups with few OTUs were dominated by invasive species (Amphipoda = 5, Blattodea = 1, Collembola = 9, Isopoda = 2, Myriapoda = 10). With the exception of Orthoptera (9) and Neuroptera (9), we recovered 40 or more OTUs from each order that was dominated by native species. The proportion of OTUs among the different orders was significantly correlated with the proportion of actual species known from the Hawaiian Archipelago for these orders (Fig. 4A,B, R^2^ = 0.710, *P* < 0.05).

The number of recovered OTUs varied significantly between collection sites (χ^2^ test, *P* < 0.05; Fig. 4C). The lowest number of OTUs was found in the youngest site (Olaa = 103, see Fig. 2 for ages). This number increased on the second youngest site (Laupahoehoe_ = _181), remained comparable in Maui (Waikamoi = 179), increased slightly on Molokai (Kamakou = 197) and then dropped marginally on the oldest site on Kauai (Kokee = 177), leading to a S-shaped, or slightly hump-shaped, diversity distribution. A slightly different picture emerged for the number of single site endemic OTUs (*n* = 473). Their number steadily increased from the youngest to the oldest site (χ^2^ test, *P* < 0.05) (Fig. 4C). While we found a considerable jump in the number of endemic OTUs between the two youngest sites (Olaa = 36, Laupahoehoe = 79), the increase gradually slowed down with increasing substrate age (Waikamoi = 109, Kamakou = 122, Kokee = 127).

**Figure 5:**
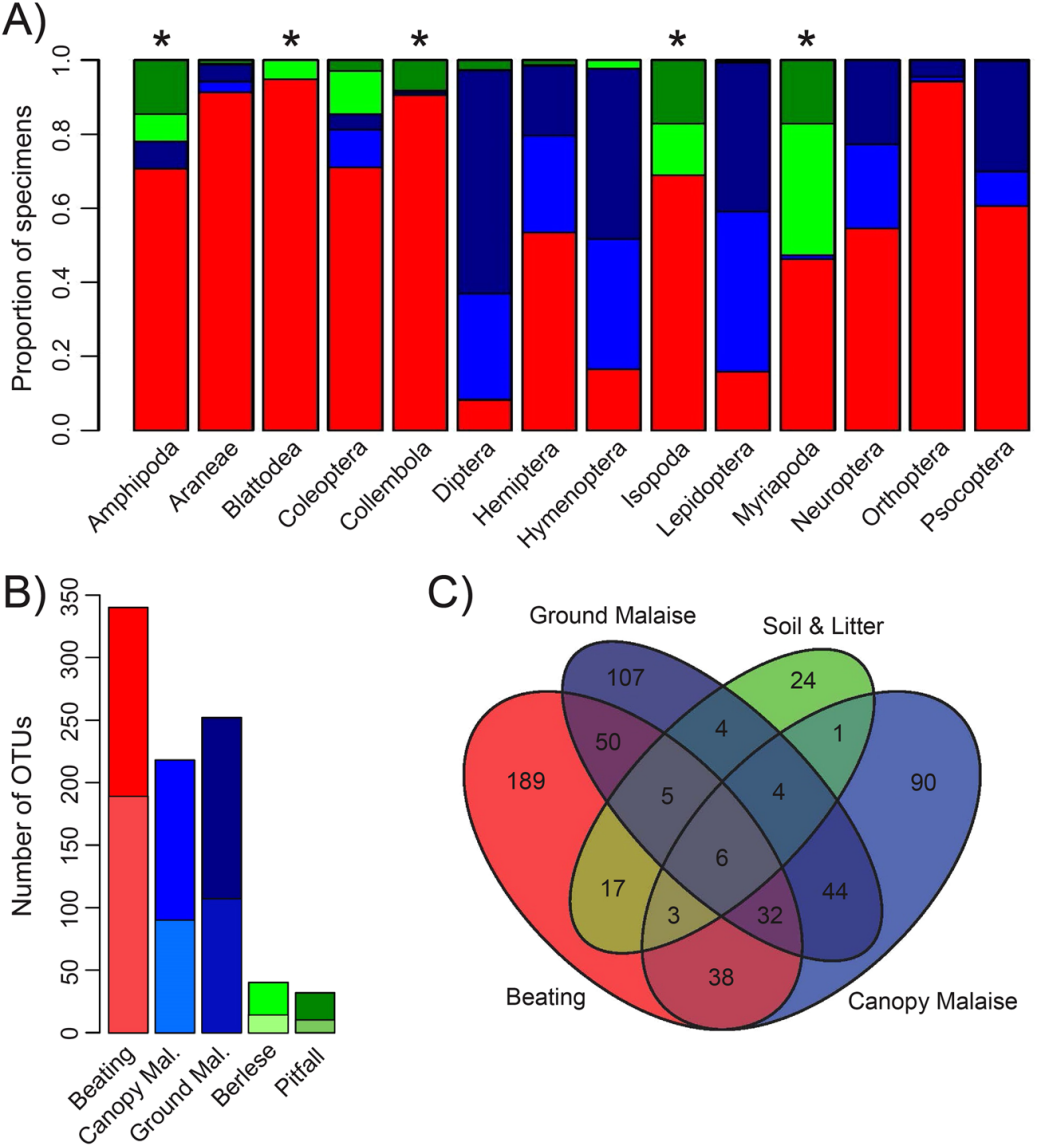
(**A**) Proportion of all 3,973 analyzed specimens from 14 orders that were collected by different trapping methods (red = beating, dark green = pitfall, light green = Berlese, dark blue = ground Malaise, light blue = canopy Malaise). A * indicates orders dominated by invasive taxa. (**B**) Number of OTUs collected by the five trapping methods. The lower, lighter colored part of each bar indicates the number of OTUs exclusively found by that trapping method. (**C**) Venn diagram showing the number of OTUs exclusively collected by separate trapping methods and overlap between methods (same colors as A, Berlese & Pitfall traps were merged).

Significantly distinct proportions of arthropod orders were collected by different trapping methods (χ^2^ test, *P* < 0.05; Fig. 5A). Pitfall and Berlese traps generally recovered fewer specimens (Berlese: 134 specimens in 10 orders, pitfall: 107 specimens in 9 orders) and were dominated by invasive taxa (60/134 for Berlese, 66/107 for pitfall traps). Malaise traps collected the majority of Diptera (441 of 496), Lepidoptera (378 of 453) and Hymenoptera (143 of 176) specimens and a considerable number of Hemiptera (204 of 453), Psocoptera (177 of 452) and Neuroptera (10 of 21). Beating yielded the most diverse collections.. The majority of specimens for 10 of the 14 orders were collected by beating and many orders were almost exclusively recovered by beating (≥90% of specimens for Araneae, Blattodea, Collembola & Orthoptera).

Different trapping methods also recovered significantly distinct groups of OTUs (χ^2^ test, *P* < 0.05) (Fig. 5B,C). Three-hundred and forty out of the 614 OTUs were found by beating, 218 by canopy Malaise traps, 252 by ground Malaise traps, 40 using Berlese and 32 using pitfall traps. Most of the OTUs found by Berlese and pitfall traps were shared with one of the other methods (40/64). In contrast, the overlap was fairly low for beating and Malaise traps. A considerable number of OTUs were exclusively collected with either one of these methods (beating = 189, canopy Malaise = 90, ground Malaise = 107). A similar pattern emerged when separate orders of arthropods were analyzed (Suppl. Fig. 3; Fisher’s exact test, *P* < 0.05). For the six most speciose orders, beating recovered most OTUs in Araneae (63/68), Coleoptera (56/84), Hemiptera (63/94), Psocoptera (34/40), and even Lepidoptera (61/112), while most Diptera OTUs (92/134) were collected by canopy Malaise traps.

### Experiment 2 – Generation of multilocus data from community samples

While each of the nuclear markers showed a relative balance in read coverage, on average we recovered significantly more reads for H3 (8,995 reads/sample) and 18S_V6-7 (8,606 reads/sample) than 28S (6,021 reads/sample) and 18S_V1-2 (5,263 reads/sample) (Pairwise Wilcoxon test, *P* < 0.05; Suppl. Fig. 4A).

**Figure 6:**
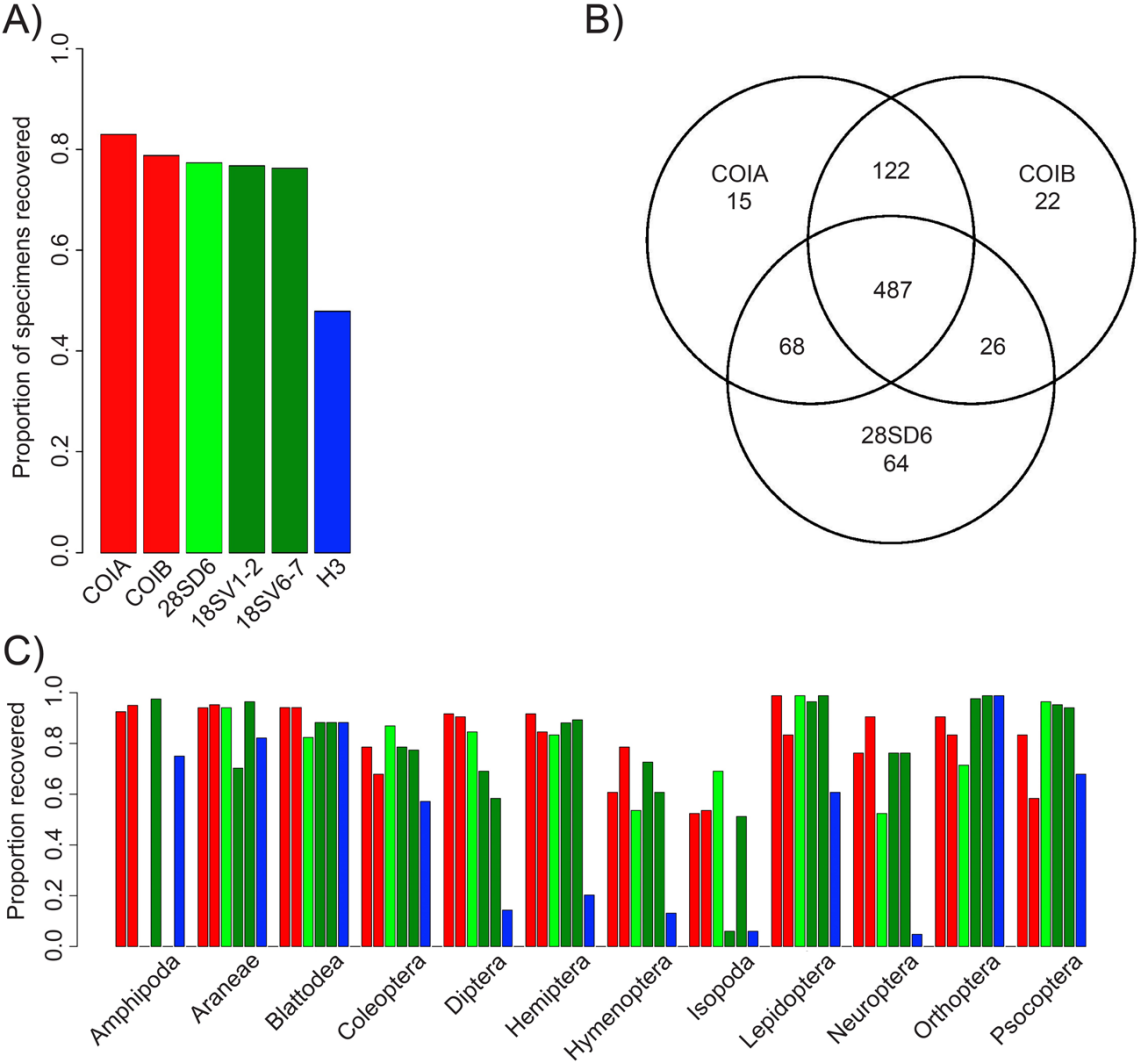
(**A**) Overall proportion of 834 arthropod specimens recovered for six different markers. (**B**) Number of recovered specimens (out of 834) for the two COI markers (COIA & COIB) and 28SrDNA and the overlap between each of these markers. (**C**) Proportion of specimens recovered for six different amplicons, separated by arthropod order.

With the exception of H3, which recovered significantly fewer specimens (49%), each marker recovered about 80% of the specimens on average (Fig. 6A, χ^2^ test, *P* < 0.05). A significant increase of recovered specimens was found by combining markers (Fig. 6B, χ^2^ test, *P* < 0.05). A combination of the two COI fragments led to a recovery of 88.73%, the combination of the two mitochondrial markers and 28SrDNA to 96.4%, and combinations of four, five or six markers led to an additional increase (97.48%, 97.84% and 98.20%, respectively). The recovery of specimens was significantly biased between taxonomic groups and loci (Fig. 6C, χ^2^ test, *P* < 0.05). Hymenoptera and Isopoda were much less well recovered than other orders, a pattern that held up for all loci. For most other orders, different loci recovered different taxa at varying efficiency.

We found considerable differences of similarity to database sequences between markers (Pairwise Wilcoxon test, *P* < 0.05) (Suppl. Fig. 4B). The two 18 S fragments showed the highest average similarity to database sequences (18SV1-2 = 98.71%, 18SV6-7 = 98.72%), followed by 28S (94.52%) and H3 (94.36%). The lowest average similarity was found for the two COI fragments (90.84 & 91.52%).

### Utility of nuclear rDNA for taxonomic assignments

For a total of 453 specimens, COI and all three rDNA amplicons were recovered. The average number of recovered OTUs for this dataset was considerably smaller for rDNA markers than for COI (Suppl. Fig. 5A). An OTU clustering at 3% similarity threshold yielded 136 OTUs for COI, 50 for 28S, 37 for 18SV1-2 and 45 for 18SV6-7. The OTU numbers of COI and the rDNA markers for different orders were significantly correlated (i.e., all these markers support the same trends of diversity across taxa; Suppl. Fig. 5A, R^2^_COI|28S _ = 0.792, R^2^_COI|18S_ = 0.817 & 0.597, *P* < 0.05). When using no OTU clustering threshold for the rDNA markers, the number of unique sequences was more similar to the actual number of OTUs for COI, especially for 28 S (Suppl. Fig. 5B, N_OTUCOI_ = 136, N_uniq28S_ = 127, N_uniq18SV1-2_ = 100, N_uniq18SV6-7_ = 91). This trend held up when separate orders were compared. Also, the number of COI OTUs was significantly correlated with the number of unique sequences for rDNA (Suppl. Fig. 5B; R^2^_COI|28S_ = 0.894, R^2^_COI|18S_ = 0.865 & 0.949, *P* < 0.05).

## Discussion

### Feasibility of large-scale analyses of whole arthropod communities

Here we show that large scale multilocus barcoding is feasible at a fraction of the sequencing cost and workload compared to current amplicon sequencing protocols^4,6^. We achieve these improvements by using DNA extractions and PCRs with pre-sorted specimen pools, multiplex PCR of different amplicons, and two levels of indexing during library preparation (see Fig. 1). Using this approach, libraries for a four-locus dataset for 100 specimens can be generated with just 12 PCRs and two clean up reactions. Compared to commonly used amplicon sequencing protocols^4^, this approach allows an up to 100-fold reduction in cost and workload. This way, DNA barcode reference libraries could be rapidly generated for unstudied ecosystems. The simple generation of multi locus data, while being able to link actual specimens to sequences, also considerably improves the taxonomic and phylogenetic resolution of DNA barcoding.

It should be noted that we here used a parataxonomic^23^ approach to characterize the DNA barcode diversity of Hawaiian arthropod communities. Identified OTUs and morphotypes do not necessarily represent actual species. A detailed taxonomic treatment of collected specimens will be necessary to conclude on actual species diversity. We are fully aware of this drawback and are currently working on the generation of a barcode reference library for actual species in our sampling sites. Our generation of a comprehensive overview of multi-locus DNA barcode data for a site will be of great value for this future taxonomic exploration. Also, we have extensively tested the targeted loci in previous work for their taxonomic resolution and suitability to assign different taxonomic levels^13,16,18^. The arthropod diversity of tropical ecosystems in greatly understudied. Considering the dwindling numbers of professional taxonomists, this situation is unlikely to be remedied in the near future. The generation of barcode data for all morphological variation at a site can aid taxonomists in selecting samples from large collections, which warrant further taxonomic treatment.

### Possible optimizations of the protocol

There are several modifications to our protocol that would allow it to be less destructive to samples and/or less expensive. In terms of the destructiveness, because of our goal of testing for an effect of body size on read content, our protocol involved extracting DNA by grinding whole bodies of differently sized specimens. However, for many studies such quantification will not be necessary, and the majority of a specimen’s morphological integrity can be maintained by subsampling tissue and combining an approximately equal mass of tissue for each specimen in the pool. This would also result in a much more even distribution of DNA per specimen, and enable sequencing at a reduced coverage. Generally, an even sampling of biomass for different specimens in a pool is recommended to achieve a better recovery of small taxa. Non-invasive DNA extraction protocols could also be utilized^31,32^ to preserve specimens for morphological analyses and/or vouchering purposes. This would be particularly important for further taxonomic explorations of the DNA-barcoded specimens.

In terms of further reducing costs, it should be possible to increase the number of pooled specimens during extractions and PCR amplification steps. Pooled specimens do not necessarily need to belong to divergent orders. Depending on the available reference databases, the resolution of barcode sequences could allow for different families, genera, or even species within a genus, to be distinguished. If specimens need to be kept separate during DNA extractions, pooling can also be performed using DNA extracts at the PCR step.

When utilizing a pooling approach, care needs to be taken to account for the possibility of the presence of parasitoids (insects whose larvae develop inside a host species). Parasitoids are particularly common among species of Hymenoptera and Diptera, thus in some cases it might not be possible to distinguish whether the barcode sequence belongs to the morphologically sorted specimen or a cryptic parasitoid larva. However, this problem can be avoided if DNA extractions are performed from legs only (i.e., avoiding the tissue of parasitoid larva in the body cavity of the host). Another problem with our approach is that the proper recovery of taxonomic identifications for specimens requires the use of reference libraries, which for many organisms and geographic localities (particularly species rich taxa in tropical regions) are currently incomplete. Those libraries that exist are known to contain numerous false identifications^33,34^. Thus, recovered sequence IDs need to be carefully cross-evaluated before assigning them to a specimen, e.g., by phylogenetic matching against a curated database or by a follow up analysis by someone with taxonomic expertise for a particular group. Moreover, PCR primers need to be carefully chosen to achieve a good recovery of a broad range of taxa. The primers we used here were previously tested and optimized for arthropods^16^. Yet, they still show biased recovery for certain groups, as e.g. seen in the limited recovery of Hymenoptera. In some cases, primers may have to be redesigned or different primers chosen for the analysis.

### Multilocus community barcoding and the utility of rDNA for arthropod taxonomy

We found that the use of multiple amplicons considerably improved taxon recovery and enabled more accurate diversity estimates. In particular, nuclear 28S ribosomal DNA emerged as a useful addition to mitochondrial COI^18,35,36^. Similar results were found in an analysis of diverse Malaise trap content using multiple COI primers^15^. Even though rDNA is considerably more conserved^37^, the recovered number of rDNA OTUs was well correlated with that found for COI. When we refrained from OTU clustering and used unique sequences, 28S supported almost the same cluster number as COI (28S = 127 vs. COI = 136). Thus, using zero radius OTUs for rDNA markers may be an advisable strategy to complement COI data with nuclear information^18^.

### Optimal arthropod collection methods

Different trapping methods strongly affected successful DNA barcode recovery. Considerably fewer specimens were recovered from soil trapping methods. Both Berlese and Pitfall traps are passive methods and expose specimens to long periods of suboptimal storage during the collection process. Yet, we noted good specimen recovery from Malaise traps, which are also passive. A major difference, however, is that for soil trapping methods, collections also result in considerable amounts of soil and debris being mixed with the samples, which can contain compounds that include PCR inhibitors^38^. Water in the soil also could have contributed to reduced yields as it dilutes the collection medium and thus possibly speeds up DNA degradation.

Besides their different utility in preserving specimens for molecular analysis, different trapping methods also showed varying efficiencies in recovering taxonomic diversity. Few taxa were exclusively found with Berlese or Pitfall traps. Moreover, the recovered soil arthropod fauna was dominated by invasive species, supporting recent findings from other oceanic islands in which the soil fauna is dominated by invasive Collembola species^39^. However, it should be noted that we excluded mites from our analysis, which can be abundant in Berlese and Pitfall traps. As soil mite communities can be highly diverse, we may have omitted a considerable proportion of soil diversity in this analysis. Interestingly, we found that the largest proportion of taxa, including many flying species, were exclusively recovered by beating. Therefore, for quick exploratory analysis aimed at maximizing the recovered diversity, we recommend beating as an efficient method of collection. Exhaustive beating of vegetation at a site can be finished within a few hours and completed within a single visit. In contrast, passive sampling devices, like Malaise traps, have to be set up at a site, left in the field and picked up again. For remote tropical ecosystems, it can take a very long time just to reach a site, leading to a considerably increased sampling effort of passive sampling over active beating. Yet, to achieve an exhaustive recovery of taxa at a site, a combination of approaches is necessary, particularly one that combines beating and Malaise traps. The latter will be particularly important to recover many Dipteran, Hymenopteran and Lepidopteran taxa.

## Conclusions

Our study highlights an approach that allows multilocus barcoding of all arthropod taxa present within a given ecosystem at minimal cost and effort. Being able to link barcode sequences to actual specimens is an advantage over community metabarcoding. The quick characterization of all DNA-barcode diversity at multiple sites is an important step towards understanding how communities have changed over time, and how they might be expected to undergo a state shift upon reaching a given “tipping point”^1^. Our method can also provide an aid for taxonomists, allowing a “reverse barcoding approach”^40^ to identify lineages in large collections, which warrant further morphological analysis. Last, we highlight beating of vegetation as a highly efficient method to maximize the recovered diversity of arthropod collections.

## Supplementary information

Supplementary Information

## Data Availability

The following data will be uploaded to the Dryad repository after acceptance of the manuscript. 1. All raw read files. 2. Alignments of all markers for all taxa. 3. Detailed sample information for all specimens. 4. The python script to assign taxonomy to BLAST result files.
