# Supplementary material for: Rapid and cost-effective generation of single specimen multilocus barcoding data from whole arthropod communities by multiple levels of multiplexing
Source: Sci Rep. 2020 Jan 9;10:78. doi: 10.1038/s41598-019-54927-z (PMC6952404; doi:10.1038/s41598-019-54927-z)
Supplement: Supplementary file 1 — Supplementary Information [file 41598_2019_54927_MOESM1_ESM.docx]

**Supplementary Material**

**Rapid and cost-effective generation of single specimen multilocus barcoding data from whole arthropod communities by multiple levels of multiplexing**

Guillemette de Kerdrel^1^, Jeremy C. Andersen^1^, Susan R. Kennedy^2,3^, Rosemary Gillespie^1^, Henrik Krehenwinkel^1,2^

1: Department of Environmental Sciences, Policy and Management, University of California Berkeley, Mulford Hall, Berkeley, California, USA

2: Department of Biogeography, Trier University, Trier, Germany

3: Okinawa Institute of Science and Technology

Corresponding author: Henrik Krehenwinkel

[Krehenwinkel@uni-trier.de](mailto:Krehenwinkel@uni-trier.de)

+49-162-9412979


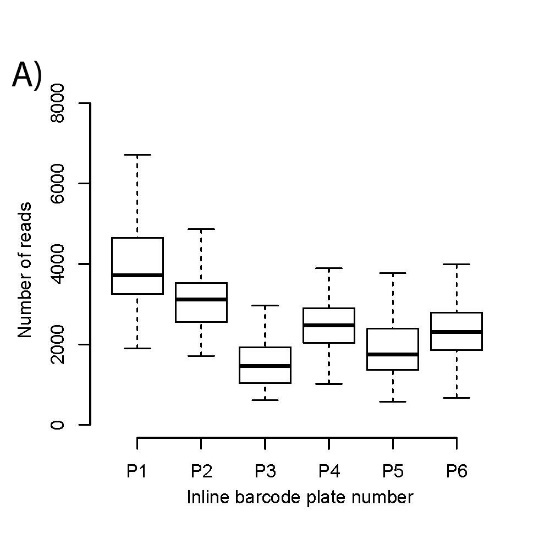

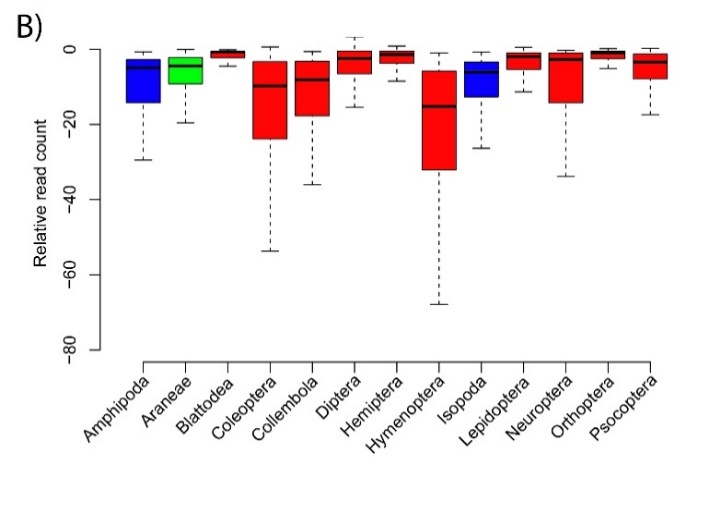

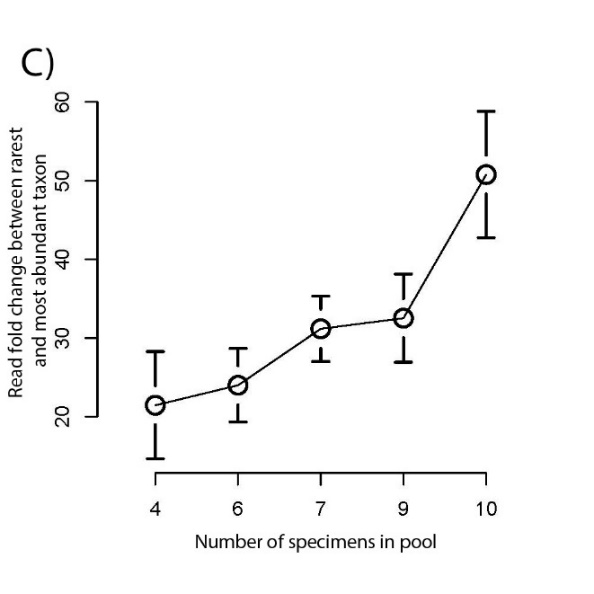


Supplementary Figure 1 A) Recovered number of reads from each well and across six different inline barcoded plates. B) Relative read abundance (fold change compared to a 1:1 association of taxon vs. read abundance) of specimens belonging to 14 different arthropod orders. C) Average fold change of read number between the most abundant and the rarest taxon’s sequence in comparison to the total number of specimens in the multiplex.


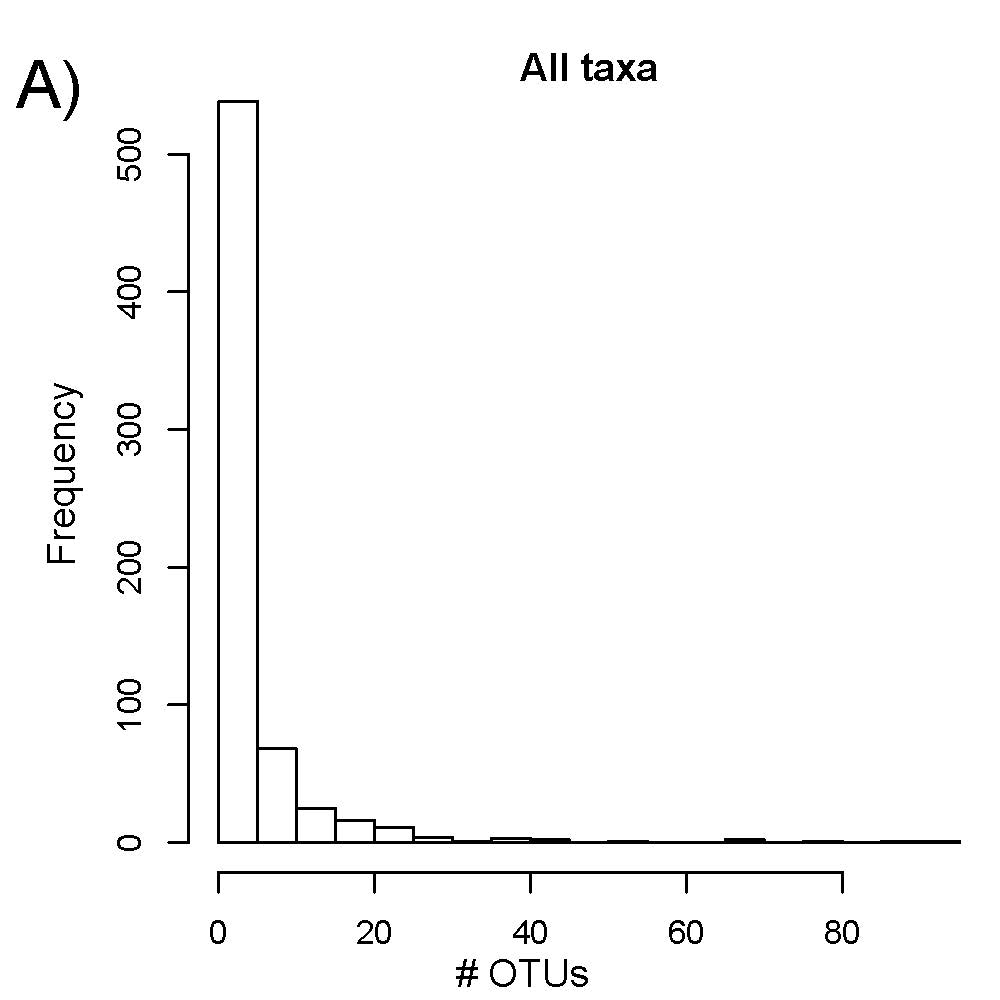

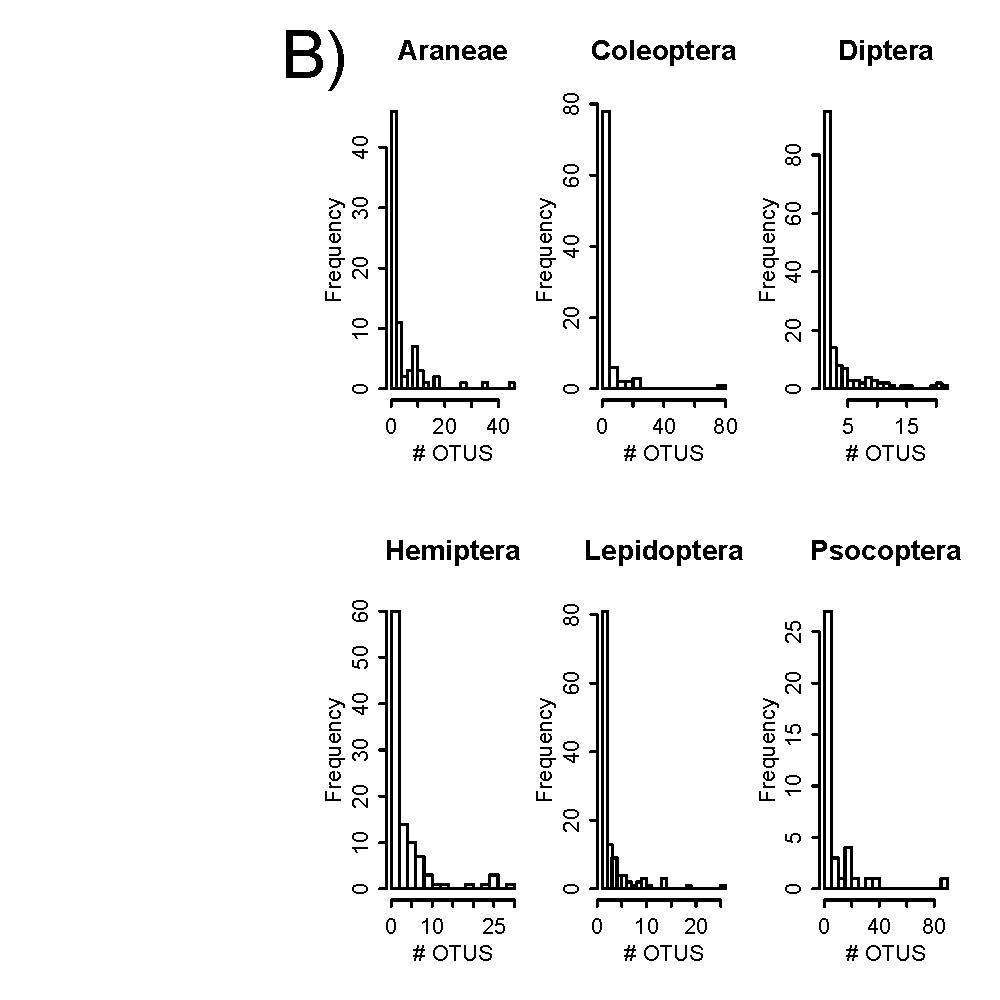


Supplementary Figure 2 Abundance distribution of the recovered OTUs for A) All arthropod specimens combined and for B) the six most speciose arthropod orders.


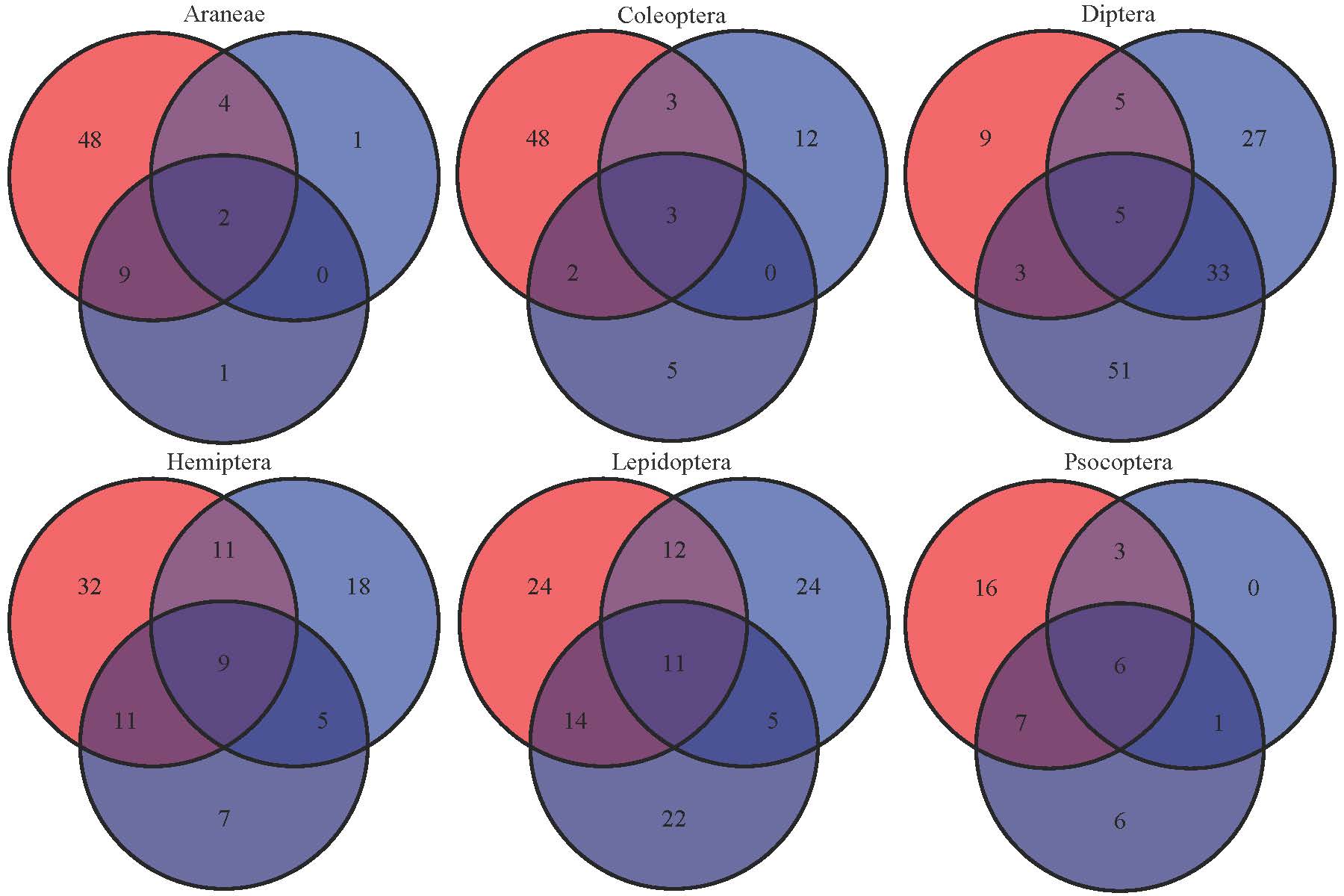


Supplementary Figure 3 Number of OTUs for the six most speciose arthropod orders, which were collected by three trapping methods (red =beating, bottom dark blue = canopy Malaise, upper right light blue = ground Malaise) and the overlap between trapping methods.


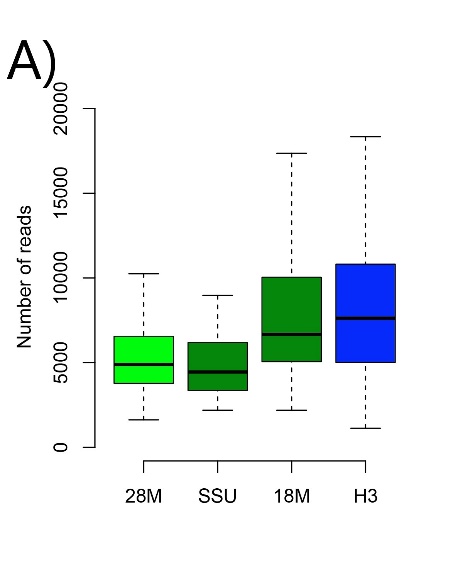

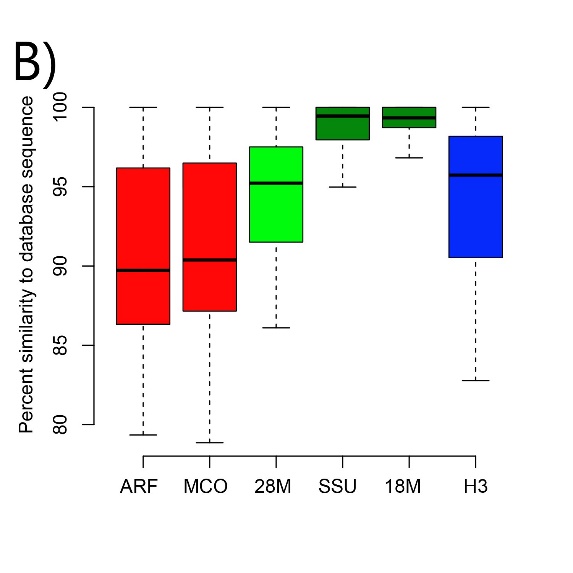


Supplementary Figure 4 A) Read coverage for every marker in the multiplex PCR. B) Percent identity of recovered sequences for the six amplicons to their most similar NCBI database sequences.


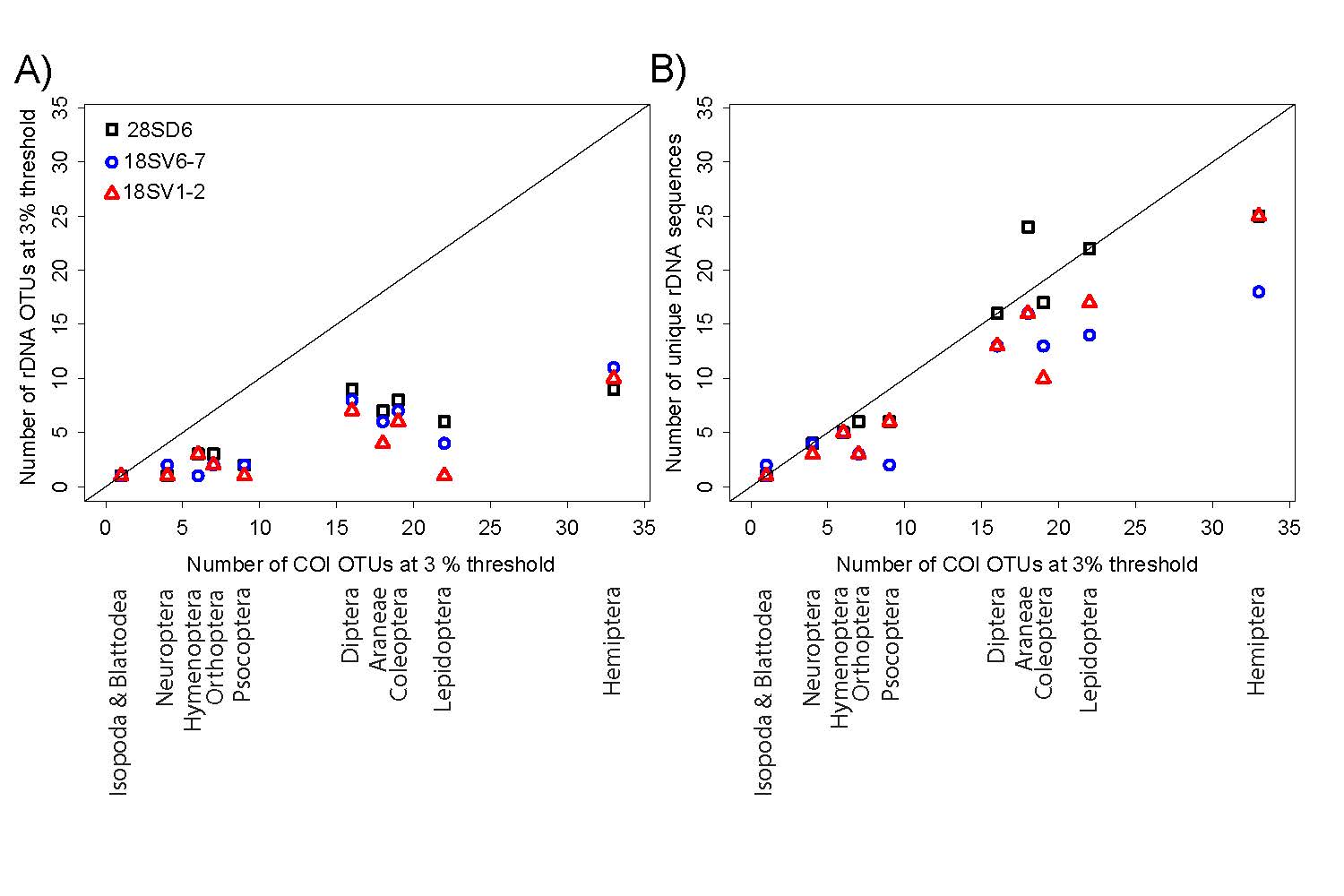


Suppl. Figure 5 A) Association of the number of recovered OTUs for different arthropod orders in our dataset of mitochondrial COI on the X-axis and the three nuclear ribosomal DNA fragments on the Y-axis B) The same association, but using the number of unique sequences for ribosomal markers on the Y-axis. The black line represents the 1:1 line for COI OTUs.
